# Sero-epidemioloical survey on African horse sickness virus among horses in Khartoum State, Central Sudan

**DOI:** 10.1186/s12917-018-1554-5

**Published:** 2018-08-01

**Authors:** Siham T. Karamalla, Ahmed I. Gubran, Ibrahim A. Adam, Tamadur M. Abdalla, Reem O. Sinada, Eltahir M. Haroun, Imadeldin E. Aradaib

**Affiliations:** 1 Molecular Biology Laboratory (MBL), Department of Clinical Medicine, Faculty of Veterinary Medicine, University of Khartoum, P.O. Box 32, Khartoum North, Sudan; 2 Scientific Research Directorate, Al-Mughtaribeen University, Khartoum, Sudan; 3 EBH Scientific Research Laboratory, Zamzam University College (ZUC), Khartoum, Sudan

**Keywords:** Epidemiology, Survey, equines, AHSV, ELISA, Sudan

## Abstract

**Background:**

African horse sickness virus (AHSV) is an infectious non contagious insect-transmitted double-stranded (ds) RNA orbivirus of the family *Reoviridae*. AHSV causes an often fatal hemorrhagic infection with high mortality among selected breeds of Arabian horses. This study was conducted to avail some information with regard to the prevalence and associated risk factors of AHSV among ecotype breeds of horses in central Sudan.

**Methods:**

Sera were collected from 320 horses, which were selected randomly from four localities and employed in the study. A competitive enzyme-linked immunosorbent assay (cELISA) was used to screen sampled sera for AHSV-specific immunoglobulin G (Ig G) antibodies.

**Results:**

Seropositivity to AHSV Ig G was detected in 275 out of the 320 horse sera, thus accounting for a prevalence rate of 85.9%. Potential risk factors to AHSV infection were reported to be associated with horse breed (OR = 5.0, CI = 0.07–2.104, *p*-value = 0.039) and activity of the horse (OR = 3.21, CI = 0.72–1.48, *p*- value = 0.008).

**Conclusions:**

The high prevalence of AHSV in Khartoum State of Central Sudan necessitates the need for continuous surveillance for AHSV infection to prevent a possible disease outbreak in this region of the African continent.

## Background

African horse sickness (AHS) is an acute disease of horses, which most often results in death of the infected animal. The disease is caused by the African horse sickness virus (AHSV), a member of the orbivirus genus in the *Reoviridae* family [1, 2]. In other equine species such as donkeys and mules, infection with AHSV is usually subclinical [3–5]. However, infected mules can amplify the virus and became seroconverted and thus play an important role in the epidemiology of the disease [6]. Nine serotypes of AHSV designated as AHSV-1 to AHSV-9 are recognized worldwide differentiated by serum neutralization test and molecular based assays [7]. An outbreak of AHS in a disease free region would have catastrophic effects on equine welfare and industry, particularly for international events such as the Olympic Games [8]. In areas of endemicity, AHSV is of concern to wildlife managers as an outbreak of the disease is likely to occur among the populations of zebra [9]. In addition, international trade of race horses is restricted to AHSV-free animals only [10]. AHSV is transmitted to horses by *Culicoides* midges, in particular by *Culicoides imicola* [11]. AHSV is related to bluetongue virus (BTV), epizootic hemorrhagic disease of deer virus (EHDV) and palyam serogroup of orbiviruses [11]. Whereas BTV, EHDV and Palyam viruses cause clinical diseases in ruminants, AHSV is mainly a disease of equines. AHSV induced a fatal infection in race horses in North Africa, the Middle East and different parts of the Arab world including the Sudan [12–14]. Serological evidence of AHSV infection in horses and donkeys is wide spread in the Sudan [6, 10, 12]. Multiple outbreaks and sporadic cases of AHSV were reported in Arabian horses in various states of the Sudan based on clinical presentation. However, the virus was isolated, for the first time, from whole blood and spleen of a mare in Khartoum State, Sudan [12]. Subsequently, AHSV was isolated from blood of infected horses in suckling mice (Aradaib, unpublished data). The two virus isolates were identified as AHSV serotype 9 (AHSV-9). Subsequently, sporadic cases and multiple outbreaks of AHSV were reported in different localities of Khartoum States. However, virus isolation attempts from blood and tissues of infected horses were largely unsuccessful.

Currently, the disease is diagnosed by conventional virus isolation, serology and molecular-based assays [15–19]. Virus isolation is tedious, time consuming, labor intensive and expensive [4, 19]. Serology is useful in epidemiologic studies to identify previous AHSV infection by detection of Ig G-specific antibodies or by detection of Ig M for detection of recent viral infections [20]. Several molecular-based assays were developed and evaluated for detection of AHSV serogroup and serotypes [21–26]. In previous studies, we have reported on a simple, rapid, sensitive and specific RT-PCR-assay for detection of AHSV serogroup in cell culture [6]. Subsequently, a more sensitive nested RT-PCR assay was also developed and evaluated for detection of AHSV RNA in a variety of clinical samples [10]. Currently, no information is available regarding the prevalence of AHSV or the potential risk factors associated with the disease among horses in Sudan. Therefore, the control of emerging viral pathogens, such as AHSV, is urgently needed in the country. The Sudan has a large numbers of horses, which play an important role in horse races and transport in remote areas. We believe further epidemiologic surveys including implementation of improved surveillance would be necessary to prevent further spread of the disease and to combat this important viral pathogen. The objectives of the present investigation were to determine the prevalence and identify risk factors associated with AHS among horses in Khartoum state, Central Sudan.

## Methods

### Study area

**Fig. 1:**
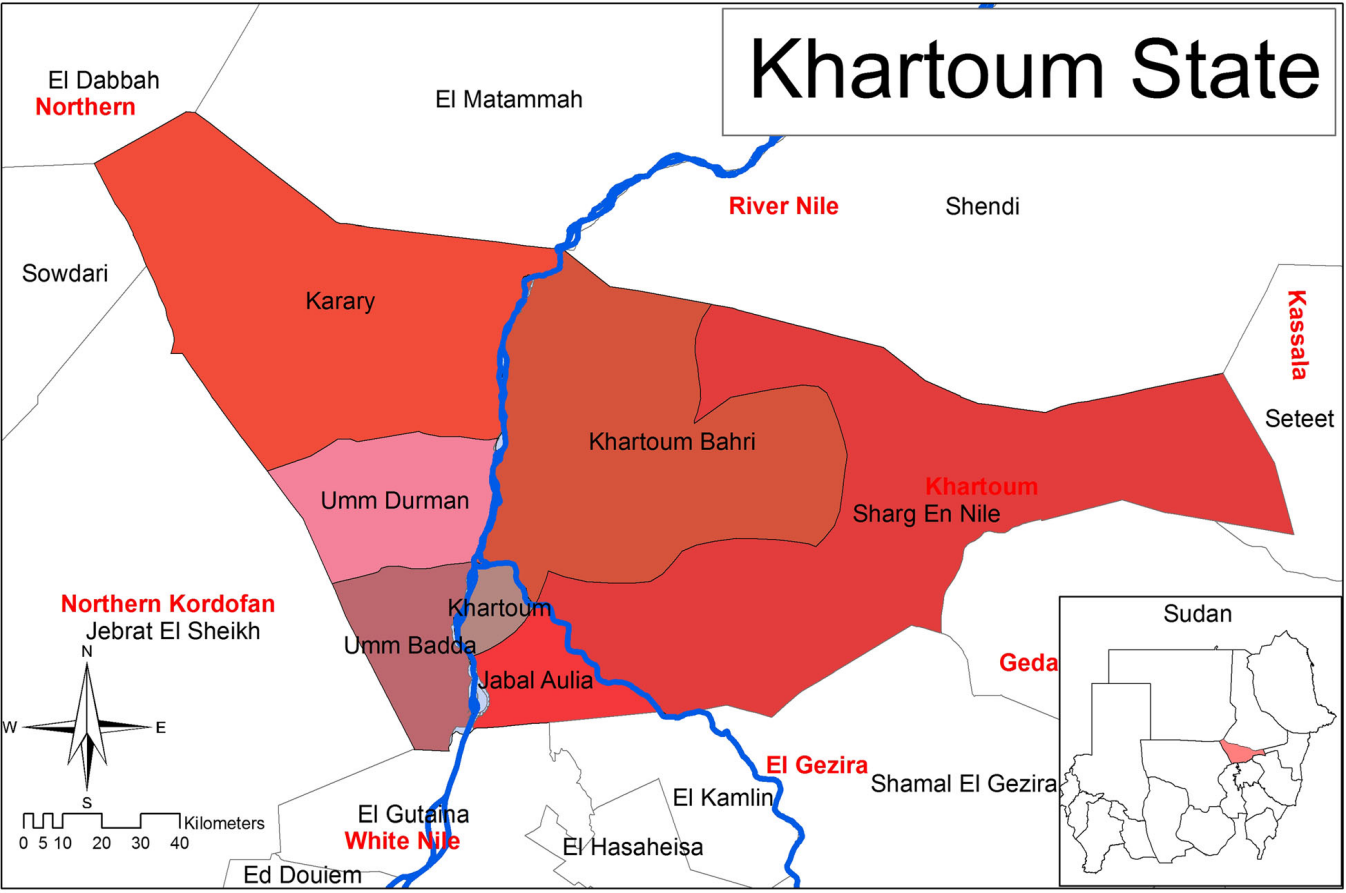
A map of the localities included in the study area of Khartoum State, Sudan

Khartoum State is one of the largest states in Sudan, which comprises three major cities. These cities are Khartoum, Khartoum North and Omdurman. Khartoum is the capital of Sudan and it is located in the middle of the country. The state covers an area of approximately 23,000 km^2^ (km^2^). The population of Khartoum state is composed of trips from different parts of the Sudan and estimated to be nearly 6 millions. The state is located at the junction of the White Nile and the Blue Nile. In Khartoum state, the 2 Niles unite to form the river Nile, which runs to the north throughout Sudan and Egypt. The state lies between longitudes 31.5 to 34 °E and latitudes 15 to 16 °N. The weather is very hot and dry in the summer season but cold dry in the winter season. Average rainfall reaches 150 mm in the north-eastern areas and 250 mm in the northwestern areas. The temperature in summer may reach up to 48 °C from April to June. In the winter, the temperature eventually declines to reach 15 °C between November and January. The horse population of Khartoum State is 6585 as estimated by the Sudan Ministry of Animal Resources, 2006 [9]. A map of Khartoum State representing the different localities is illustrated in (Fig. 1).

### Study design

The present investigation was conducted as a cross sectional survey in Khartoum State of the Central Sudan. A multistage probability sampling method was applied in this study. Four localities in Khartoum state including Omdurman, East Nile, Bahri and Khartoum were selected randomly. Eight herds in each village of the four localities were selected. Finally, 10 blood samples were collected from horses in each herd using simple random sampling method, which accounts for a total of 320 blood sample size [27]. Horses over one year of age were considered for the purpose of this study to avoid detection of maternal AHSV antibodies in young animals.

### Questionnaire

A structured questionnaire was employed for the survey and was applied to all animals participated in the study. The information required for the survey was obtained from the animal owners. Individual and management risk factor attributes were included in the questionnaire. The individual risk factors including age (younger animals < 2 years older animals 2 years and above), sex (male, female), breed (through breed, local breed), body condition (fair and thin); and the management risk factor attributes including insect vector (presence or absence), insect control measures (practiced or not), activity of the horse such as chaser (race horse), cart horse involved in transport of materials, and police horse involved in security of the state by the police at night. In addition, the four localities were also included in the study.

### Ethics approval and consent to participate

The purpose of the study was made clear to the animal owners participated in the study. Those who agreed to participate in the study were requested to provide a written consent. All the information related to the AHSV risk factors was obtained from the animal owners. The ethical clearance for the study was obtained from the Research Board of the Faculty of Veterinary Medicine, University of Khartoum, Sudan.

### Collection of blood samples

Blood samples were collected from horses by well trained veterinarians to ensure proper physical restraint of animals to avoid injury. Blood samples were collected from the jugular vein of the selected horses in the study area of Khartoum State, Central Sudan. A total number of 320 sera were collected randomly from the animals and employed in this study. Blood samples were allowed to clot and sera were separated and kept frozen at − 20 °C until used for screening of horses for seropositivity to AHSV-specific IgG antibodies using competitive indirect enzyme-linked immunosorbent assay (cELISA).

### Enzyme-linked immunosorbent assay (ELISA)

Detection of AHSV-specific Ig G antibodies was made possible using a commercially available cELISA kit (Ingezim AHSV, Compact Plus, Spain). The cELISA was performed in antigen coated plates. A total of 100 μl (μl) test volumes were used in the cELISA assay. All incubations were performed for I h at 37 °C and washing of plates was performed three times with the provided washing buffer. The results of the cELISA were recorded using ELISA reader set at 630 nm. The test sample was considered positive when the optical density is less than 50% of the mean of the negative controls.

### Statistical analyses

Statistical package for social sciences (SPSS) software package for window (version 21.0) was employed to enter the data in the computer. The univariable analysis using Chi-square (χ2) test was used to determine the associations between the outcome variable (status of AHSV seropositivity in horses) and its potential risk factors. Significant association between AHSV and risk factor was initially considered when *P* value < 0.25 (two tailed; α = 0.25). The results of the univariable analysis were further subjected multivariable analysis using logistic regression. Results were expressed as odd ratios (OR) with 95% confidence intervals (C.I) for each risk factor. *P* value less than 0.05 (*p* < 0.05) represents significant association between the AHSV seropositivity and associated risk factors.

## Results

AHSV seropositivity was recorded in 275 out of the 320 horses as detected by the cELISA, which accounts for prevalence rate of 85.9%. The highest rates of AHSV seropositivity was recorded in East Nile locality (94%) whereas the lowest rate of seropositivity was recorded in Bahry locality (81.5%). The univariate analysis using Chi-square test was conducted for the association between the potential risk factors and AHSV seropositivity. The initial results using univariate analysis showed that the independent variables including, body condition, breed, activity of the horse, presence of insects and locality were statistically significant *p* > 0.25 (Table 1). The significant results in the univariable model were further re-entered into final multivariate model using logistic regression analysis to illuminate confounding factors. A *p*-value < 0.05 was considered statistically significant. Horse breed (OR = 5.0, CI = 0.07–2.104, *p*-value = 0.039) and activity of the horse (OR = 3.21, CI = 0.72–1.48, *p*- value = 0.008) were recorded as potential risk factors for contracting AHSV infection. The results are summarized in (Table 2). The rest of the risk factors did not show any significant association with AHSV seropositive horses.

**Table 1:** Univariate analysis for the association between potential risk factors and AHSV infection among horses in Khartoum state, Sudan, using chi-square test

Risk factors	Animals tested	Animals affected (%)	df	*χ*2	*p*-value
Locality			3	6.7	0.079
East Nile	50	47(94%)			
Bahry	108	88(81.5%)			
Omdurman	35	33(94%)			
khartoum	127	107(84%)			
Age			1	0.29	0.58
young	89	78 (87.6%)			
Old	231	197(85.3%)			
Sex			1	0.70	0.48
Female	50	41(82%)			
Male	270	234(86.7%)			
Breed			2	7	0.031
Arabi	14	12(85.7%)			
Cross	30	21(70%)			
Local	276	242(87.7%)			
Body condition			1	4.6	0.03
Fair	294	249(84.7%)			
Thin	26	26(100%)			
Presence of insects			1	1.94	0.16
No	33	31(93.9%)			
Yes	287	244(85%)			
Control measures			1	0.49	0.48
No	193	168(87%)			
Yes	127	107(84.3%)			
Activity			2	6.01	0.049
Chaser	73	60(82.2%)			
Cart horse	108	88(81.5%)			
Police horse	139	127(91.4%)

**Table 2:** Multivariate analysis using logistic regression model for significant association (*p* > 0.05) between risk factors and AHSV infection among Horses in Khartoum State, Sudan

Risk factors	OR	95%C I	*P*-Value
Breed			
Cross	Reference		
Arabi	5	0.07–2.104	0.039
Activity			
Cart horse	Reference		
Chaser	3.21	0.072–1.48	0.008

## Discussion

Infectious viral pathogens, such as AHSV, are reemerging as major veterinary problems in different parts of the African continent [13, 14]. Very little information is available about the epidemiology of AHSV in the Middle East and East Central Africa including the Sudan. To better predict and respond to AHSV outbreak, this cross sectional study has been initiated to estimate the prevalence of AHSV and to identify the potential risk factors associated with the disease among horses in Khartoum State of the Central Sudan. The study area of Khartoum state has been selected as it is considered the center for international trade of horses in the country. The routine surveillance for AHSV would be necessary to monitor incursion of the disease in other part of the country considered to be AHSV- free zones. The present study showed that the prevalence of AHSV seropositivity is gnificantly high (85.9%). The detection of AHSV antibodies indicated previous exposure of horses to AHSV. The high prevalence rate (85.9%), as indicted by detection of AHSV antibodies, showed an evidence of previous circulation of AHSV in the study area. There is no vaccination program for AHSV in the Sudan. Therefore, the detected antibodies were due to natural infections. It should also be noted that detection of maternal antibodies to AHSV was excluded as horses included in this study aged over one year.

The present study showed that there was a significant difference between the AHSV seropositivity rate and the breed of the horse. Exotic (Cross) breed of horses are at 5 times at risk compared to indigenous (local) breed of Sudanese horses. High seroprevalence was observed among cross breeds with high percentage of exotic blood. Exotic breeds are usually highly susceptible to bites of insect vectors and subsequent development of AHSV infection. In contrast, the indigenous breed of horses are relatively resistant to bites of insect vectors hence, they were at lower risk for the disease compared to cross breed. In addition, there was a significant difference between AHSV seropositivity and the activities of the horse, particularly those involve in horse racing. Race horses are at 3 times more likely to be at risk compared to other horses involved in transportation activities. Once again, this is attributed to the fact that almost all race horses are of the Arabian horse origin or imported from other countries. It is also possible that most race horses are not usually kept in insect-secured houses and are likely to be exposed to the insect vector as is the case in most horse houses in Khartoum State. The highest rates of AHSV seropositivity was recorded among horses in the East Nile locality (94%). This is most probably to construction of irrigation projects and agricultural schemes in this locality, which provide conducive environmental condition for the breeding of *Culicoides* midge, the insect vector. However, the lowest seropositivity rate was recorded among horses in Bahry locality (81.5), which is possibly attributed to dry climate condition and high variations in temperatures. The present study illustrated that AHSV is now becoming broadly distributed in Sudan with a very high prevalence rate of 85.9% in Khartoum state. It is recommended that the survey should be extended in the future to include virus isolation attempts and to identify the AHSV serotypes circulating in the country. Molecular epidemiological studies such as, viral sequence analysis and phylogeny, should be conducted to monitor the movement of the virus between these African countries. The rest of the individual or management risk factors did not show any significant association with HSV seropositivity. Male or female horses have no significant association with AHSV seropositivity. In addition, no significant difference was observed between AHSV seropositivity and the age of the animals. A Previous study on Seroprevalence of the disease in Ethiopia reported that age and sex were not significantly associated with seroprevalence of AHSV [14]. Early diagnosis during disease outbreak among non vaccinated equines would be necessary for prevention and control of the disease [28]. Complete viral genome sequencing and phylogeny would be necessary to determine the virus genetic lineages [29]. It should be noted that treatment of horses with insecticides be applied monthly to prevent insect bite. The animal owners should also be educated about the risks of the disease, and prevention of the infection through insect control program.

## Conclusions

The prevalence of AHSV is significantly high (85.9%) among horses in Khartoum State, Sudan. Exotic Breed and activity of the horse particularly those involved in racing activity are considered to be at higher risk of contracting AHSV infection. This study would be expected to reduce the impact of the disease on horse breeders and subsequently avoid a possible AHSV outbreak among horses. Virus isolation attempts would be necessary for subsequent molecular characterization studies. It is recommended that complete viral genome sequencing and phylogenetic studies should be conducted to determine the genetic lineages of AHSV serotypes circulating in Sudan to follow the movement of the virus in the African continent.

## Abbreviations

AHSV: African horse sickness virus
BTV: Blue tongue virus
CI: Confidence interval
EHDV: Epizootic hemorrhagic disese virus
ELISA: Enzyme-linked immunosorbent assay
HRP: Horse radish peroxidase
Ig G: Immunoglobulin G
OR: Odd ratio
RT-PCR: Reverse transcriptase polymerase chain reaction
Μl: Microliter
